# Current practice and adaptations being made for people with autism admitted to in-patient psychiatric services across the UK

**DOI:** 10.1192/bjo.2021.58

**Published:** 2021-05-14

**Authors:** Keir Jones, Satheesh Gangadharan, Philip Brigham, Edward Smith, Rohit Shankar

**Affiliations:** Intellectual Disability Department, Leicestershire Partnership NHS Trust, UK; Intellectual Disability Department, Leicestershire Partnership NHS Trust, UK; Cornwall Intellectual Disability Epilepsy Research (CIDER) Cornwall Partnership NHS Foundation Trust, UK; Autistica, UK; Cornwall Intellectual Disability Epilepsy Research (CIDER) Cornwall Partnership NHS Foundation Trust, UK; and University of Plymouth Medical School, UK

**Keywords:** Autism, intellectual disability, developmental disability, inpatients, mental health

## Abstract

**Background:**

A significant number of people with autism require in-patient psychiatric care. Although the requirement to adequately meet the needs of people with autism in these settings is enshrined in UK law and supported by national guidelines, little information is available on current practice.

**Aims:**

To describe characteristics of UK in-patient psychiatric settings admitting people with autism. Also to examine psychiatric units for their suitability, and the resultant impact on admission length and restrictive interventions.

**Method:**

Multiple-choice questions about in-patient settings and their ability to meet the needs of people with autism and the impact on their outcomes were developed as a cross-sectional study co-designed with a national autism charity. The survey was distributed nationally, using an exponential and non-discriminatory snowballing technique, to in-patient unit clinicians to provide a current practice snapshot.

**Results:**

Eighty responses were analysed after excluding duplications, from across the UK. Significant variation between units across all enquired parameters exist. Lack of autism-related training and skills across staff groups was identified, this becoming disproportionate when comparing intellectual disability units with general mental health units particularly regarding psychiatrists working in these units (psychiatrists: 94% specialist skills in intellectual disability units versus 6% specialist skills in general mental health units). In total, 28% of survey respondents felt people with autism are more likely to be subject to seclusion and 40% believed in-patients with autism are likely to end in segregation.

**Conclusions:**

There is no systematic approach to supporting people with autism who are admitted to in-patient psychiatric units. Significant concerns are highlighted of lack of professional training and skill sets resulting in variable clinical practice and care delivery underpinned by policy deficiency. This could account for the reported in-patient outcomes of longer stay and segregation experienced by people with autism.

## Background

Available evidence points to a significantly greater prevalence of autism spectrum disorders in people admitted to in-patient mental health services when compared with the general population.^1,2^ A National Autistic Society report indicated a 7% increase in people with autism admitted to hospital in England between March 2015 and October 2018.^3^ This was despite the NHS England ‘Transforming Care’ programme that aimed to reduce avoidable admissions in this group. Meeting the needs of this group is particularly important within the intellectual disability population (also known as learning disability in UK health services), as between 20% and 30% of people with an intellectual disability are estimated to have comorbid autism.^4^ Historically, people with autism are more likely to have been supported within an intellectual disability setting for behavioural and mental health concerns. This is now changing with the current political focus on their behavioural and emotional needs being met by ‘main streaming’ i.e. requiring mainstream services to make reasonable adjustments to meet the needs of their patient group with autism.^5^

There is significant overrepresentation of mental health needs in people with autism.^6,7^ This is highlighted by experiences of suicidal ideation, considered to be up to nine times more common than in the general population.^8^ This population therefore is at a higher risk of psychiatric admissions, both voluntary and involuntary. The specific challenges for people with autism in terms of their communication and cognitive profile, particularly if associated with an intellectual disability, could lead to difficulties in diagnosis and treatment of their mental health needs.^7^ This has the potential to leave them vulnerable to longer admissions and at risk of institutionalisation. The requirement to adequately meet the needs of people with autism is enshrined in UK law and in the associated statutory guidance to health and social care organisations in England.^9,10^ This places a legal obligation on local authorities and National Health Service (NHS) trusts to provide adequate training, specialist services and reasonable adjustments for people with autism. However, although this has been outlined in theory in various good practice guidance^7,10^ there is little real-world evidence of implementation of these measures across the different in-patient psychiatric settings in the UK that admit people with autism.

Admission to an in-patient psychiatric facility can be extremely unsettling and frightening for anyone. Many aspects of an admission may prove more distressing and/or disorientating for people with autism.^7^ Possible challenges include the sudden environmental and sensory changes, increased and unfamiliar social and communication demands and significant change in routines.^11^ In addition to this, there is a potential lack of access to usual safe spaces and coping mechanisms particularly needed for an individual with autism. These additional challenges may explain growing evidence of increased length of stay, increased rates of distress and agitation, increased use of restrictive interventions such as ‘long-term segregation’ (a situation where, in order to reduce a sustained risk of harm posed by the patient to others, which is a constant feature of their presentation, a multi-disciplinary review and representative from the responsible commissioning authority determines that a patient should not be allowed to mix freely with other patients on the ward on a long-term basis) and seclusion (the supervised confinement and isolation of a patient, away from other patients, in an area from which the patient is prevented from leaving, where it is of immediate necessity for the containment of severe behavioural disturbance which is likely to cause harm to others) for people with autism with concurrent mental illnesses.^12–14^ A recent UK study from secure care suggested that individuals with autism experienced both a higher number of episodes and longer duration of long-term segregation than individuals without autism.^15^ A Finnish study found that people with autism were significantly more likely to experience restraint (odds ratio 4.5, 95% CI 2.0–9.9).^16^

In the UK, people with a known autism diagnosis presenting with mental health or behavioural concerns are admitted to specialised intellectual disability units although the role of local psychiatric units and other specialist facilities (forensic etc.) is increasing. However, there is little research on whether the needs specific to people with autism are being met irrespective of the setting.

## Aims

Our aims were as follows.

- To explore the skills and adaptations that current in-patient psychiatric services which admit people with intellectual disability have made to meet the needs of people with autism across the UK.
- To explore in-patient clinicians’ views on current length of stay and use of restrictive interventions for in-patients with autism in the UK based on their experience.

## Method

An online survey was developed in association with an UK autism charity between February and April 2020 and ran for 4 weeks in June/July 2020. The survey questionnaire can be found in Supplementary File 1 available at https://doi.org/10.1192/bjo.2021.58. The draft questionnaire was constructed by the authors based on a review of the literature. It was developed by peer consultation led by the us.

The survey was undertaken online using the google platform and set to take approximately 5–10 min to complete. This was felt the optimum time to balance response engagement and gain the minimum required information to draw meaningful conclusions. The survey questionnaire had 16 questions that aimed to assess clinicians’ perceptions about and approach to individuals with autism supported in psychiatric in-patient settings. The survey consisted of a mix of questions with predetermined answers, questions requiring the answer to be entered and questions that allowed for free-text comments. We collected limited demographic details from the participants, though broadly, the survey was anonymous and all results anonymised.

The principal themes of the questionnaire were:

(a) demographics and area of work;
(b) staff expertise;
(c) assessments undertaken relating to autism;
(d) adaptations, including environmental and communication tools;
(e) use of long-term segregation/seclusion;
(f) care pathways;
(g) other comments/feedback.

It was circulated using an exponential and non-discriminatory snowballing technique, commencing with key personal contacts working in in-patient psychiatric settings. These contacts were then requested to forward the link within their own professional networks. The networks included consultant psychiatrists in intellectual disability/autism; higher specialty trainees in psychiatry of intellectual disability/autism; and intellectual disability/autism nursing networks. Other relevant networks such as forensic and general mental health were reached out to by personal contact from us. This should be considered non-probability sampling, as it does not include complete coverage of services in the field and/or any particular sector.

Analysis of data was performed using Microsoft Excel. Descriptive statistical analyses were carried out primarily to provide data on proportions using SPSS version 25 for Windows. The survey had two sections. The first section looked primarily at collecting relevant demographic information and describing the availability of provision in a geographical area for people with intellectual disability/autism. The second section looked to ascertain the autism-specific expertise, adaptations, processes and outcomes within that setting,

### Ethics and participation consent

No ethical permission was required as this was a study to evaluate knowledge and attitudes as part of a service evaluation. Further, the respondents were clinical practitioners where consent was implicit by participation. All participants were advised at the start of the study that participation was voluntary and their replies i.e. data would be anonymised and analysed. We also used the NHS Health research authority tool (http://www.hra-decisiontools.org.uk/research/index.html) that helped confirm that no ethics was needed for this project (Supplementary File 2).

## Results

Overall, 90 responses were received from varied geographical regions across the UK. On reviewing the data using the postcodes provided, we identified that there were multiple responses from the same postcode for some. Where there were multiple responses from the same postcode, responses from the same postcode were counted only if they related to a different unit (an example is one response was from an assessment and treatment unit and another response from a forensic unit). If there were multiple responses from the same unit, the response with the most questions answered was chosen. Other same-unit responses were used to examine the validity of the principal responder. After eliminating duplication, we included 80 responses for further analyses.

In total, 22 responses were received from London and South East England, 18 responses from North East England and Yorkshire, 8 responses from North West England, 8 responses from the East of England, 8 responses from South West England and 7 responses from the Midlands. There were also four responses from Wales and one from Scotland. Four responses could not be placed geographically but were otherwise valid responses and hence were included in the analysis.

The clinicians responding were based in a variety of clinical settings (please see Table 1) with staff in general adult mental health units accounting for approximately a quarter of respondents and staff in intellectual disability specialist units approximately a fifth of respondents. Although the vast majority of responses were from the NHS (92.5%), four responses came from independent sector hospitals and two from units with mixed funding.

**Table 1:** Nature of in-patient unit

	*n*	%
Child and adolescent mental health services	2	2.5
Forensic learning disability unit	10	12.5
Forensic unit non-learning disability	8	10
General adult mental health unit	21	26.3
Learning disability	17	21.3
Mental health hospital	2	2.5
Mixed	12	15
Perinatal	2	2.5
Rehabilitation	3	3.8
Specialist autism unit	3	3.8
Total	80	100

Of the respondents 58.9% stated that there was access to a specialist assessment and treatment unit for people with intellectual disability/autism in the area. Nearly half (46.6%) stated that there was access to assessment and treatment within general adult mental health in-patient units. Only 11% had block commissioning of private sector beds and 22% had arrangements for spot purchase of assessment and treatment beds as required for people with intellectual disability/autism.

The proportion of people with autism being admitted to the respondent's unit varied from less than 10% (27 units) to 50% or over (18 units). Over half of the units reported that their patient group included more than 10% people with autism. Assessment and treatment and forensic units specifically catering for people with an intellectual disability/autism generally reported higher proportions of people with autism among their cohort. Of these 23 units, 11 of them reported that over 50% of their patient group had an autism diagnosis.

Staff team specialist knowledge, training or skills with regards to autism was enquired about and results are presented in Table 2. Across the multidisciplinary team, the proportion of clinicians with specialist skill sets in autism ranged from 46% to 60%.

**Table 2:** Staff expertise

Profession	*n*, (*N* = 74)	Specialist skills related to autism, %
Psychiatrist	34	46
Speech and language therapists	42	57
Occupational therapists	44	60
Nurses	41	55
Psychologist	43	58

A comparison of the spread of professionals with autism expertise across the two main in-patient settings revealed intellectual disability/autism units were better equipped than general settings (Table 3) with striking discrepancies in skill sets across the professions; starkest in the psychiatrists found in each setting (94% *v.* 6%).

**Table 3:** Comparison of staff expertise between intellectual disability-specific assessment and treatment units and general adult mental health units

Professionals with expertise in autism	*n*, (*N* = 17)	Intellectual disability units, %	*n,* (*N* = 17)	General adult mental health units, %
Psychiatrists	16	94	1	6
Speech and language therapists	15	88	6	35
Occupational therapists	16	94	3	18
Nurses	11	65	6	35
Psychologists	14	82	6	35

The survey also looked at the assessments in place for in-patient services to support people with autism in a person-centred manner as per current good practice (Table 4). In total, 90% of units reported offering autism assessment, and just over 80% has specific assessments on individual's ‘likes and dislikes’ and looking at coping strategies. Care plans tailored to the needs of the individual with autism were available in 71% of units.

**Table 4:** Additional assessments provided for patients with autism

Specific support for people with autism	*n*, (*N*)	Units providing this, %
Assessment of autism	71 (79)	90
Care plans based on individual needs specific to people with autism spectrum disorder	53 (75)	71
Sensory assessment	49 (79)	62
Assessment of likes and dislikes	64 (79)	81
Assessment of coping strategies	65 (79)	82
Communication passports	52 (79)	66
Specific protocol for admission, assessment and management of people with autism spectrum disorder	17 (79)	21

However, only two-third of units provided communication passports and just over 60% a bespoke sensory assessment. The presence of a standardised protocol for people with autism was available only in a fifth of the respondent's units. The range of communication support provided for people with autism was explored. Of all units 63% provided visual signage or orientation tools, 76% were able to provide visual timetables, 74% were able to provide visual help/cue cards and 60% were able to provide social stories.

In terms of specific adaptations beyond communication support, one of seven units (14%) reported being unable to provide any extra adaptations for people with autism. Table 5 details other autism-relevant provisions made available in the respondent's units. Other adaptations mentioned in the free-text included ear defenders, weighted blankets, stress ball and relaxing music.

**Table 5:** Additional provisions/adaptations provided for people with autism

Type of provision	*n*, (*N* = 79)	Units providing this, %
Open access low-stimulus area	41	52
On request low-stimulus area	33	42
Scheduled access low-stimulus area	12	15
Lighting adaptations	18	23
Ability to adapt meal plans to sensory requirements	40	51
Noise adaptations	11	14
Other adaptations	3	4
No adaptations provided	12	15

The experiences and outcomes for people with autism in in-patient settings from the perspective of the clinicians working there were solicited. Three proxy measures, which may reflect patient experiences or outcomes, were enquired into (Table 6). Two-thirds of units responding felt that people with a diagnosis of autism were more frequently subject to delayed discharge. Nearly a third (28%) felt that people with autism were more likely to be secluded during their stay and 40% reported episodes of long-term segregation for people with autism in the past year.

**Table 6:** Reported outcomes for in-patients with autism in comparison to other in-patients in the unit

Nature of outcome	*n*, (*N*)	Units with the outcome, %
Patients with autism likely to have discharge delays	40 (61)	66
Patients with autism more likely or significantly more likely to be secluded during their in-patient stay	22 (79)	28
Patients with autism subjected to long-term segregation in the last 12 months	30 (76)	40

## Discussion

To our knowledge, this is the first systematic survey undertaken in the UK examining the characteristics of the support available for in-patients with autism in psychiatric units. Data has been collected directly from various professionals to assess realistically how people with autism are supported. The survey provides a reflection of real-life practice, gathering the experience of ‘shop floor’ clinicians that can help focus further work to improve the in-patient experiences and management of people with autism. The survey successfully manages to bring together opinions from across the UK to help understand the challenges facing this vulnerable population with regard to in-patient support and care. Although the survey was UK wide the majority of responses were from England, thus more representative of the English nation than of the three devolved nations. However, the responses across England were across all geographical regions and proportionately well represented.

### Limitations

First, it is difficult to envisage if the participants’ responses suitably capture the quantity and indeed the quality of the units they worked in, which is a methodological limitation of the survey approach. However, there appears to be face validity in responses when the small samples of duplicate responses emerging from the same units were looked at and compared. This gives confidence in the study results. Second, it is possible that more of those who are engaged or interested in supporting people with autism have responded to the online survey compared with those who are not. This may have introduced bias in the data. Third, some questions might be perceived as ambiguous and there may be some overlap between questions. Relying on retrospective reports and answers is likely to lead to approximations. A further challenge is that different regions had different response rates. This obviously lends itself to the survey gaining a big picture as opposed to being definitive in its conclusions.

The survey method, of exponential and non-discriminatory snowballing technique commencing with key personal contacts and these individuals forwarding the link within their own professional networks, means that we cannot establish a response rate. Nor could we explore the characteristics of non-responders.

### Interpretation of our findings

In spite of the limitations, the survey has captured critical knowledge and evidence hitherto unavailable in the scientific literature. It is interesting to note that all responding units had engagement with people with autism but numbers varied. The heterogeneous approach to facilities for assessment and treatment for people with autism in different regions stands out with approximately half of the respondents suggesting access to specialised intellectual disability/autism units with the other half suggesting access to mainstream mental health units. There appears to also be a lack of a proactive procurement bed policy for this vulnerable group with only a minority of reporting areas having pre-emptive commissioning arrangements.

Given the diverse nature of the needs people with autism present with it is concerning to see that across the UK there is a significant gap in professional competencies in providing person-centred input with only 46–60% of professionals, (depending on specialism), having relevant skill sets in in-patient settings suitable for supporting people with autism. This gap in skills across professions appears to be further magnified when the focus is on general mental health units. Compellingly, thematic analysis of the associated comments for this question confirmed that the respondents had insight and awareness of this lack of skills and associated training. Particularly of concern is the significant skew in staff skills and training in supporting people with autism that we found in intellectual disability/autism units (65–94%) compared with general mental health units (6–35%). In particular, the difference between the skills of psychiatrists working in intellectual disability units relating to people with autism (94%) and those of psychiatrists in general in-patient mental health units (6%) is very worrying indeed. The gap in skills between the two settings suggests that people with autism are likely to encounter a postcode lottery to where, how and quality of mental health services to meet their needs. Even in intellectual disability units the individual professional skill sets are heterogeneous, which undoubtedly will affect care delivery.

In a similar vein the assessments, processes and interventions specific to people with autism offered in the units of respondents are mixed and diverse. The majority offer an autism assessment and most units offer a range of autism-specific interventions. There is a lack of consistency on what is on offer and likewise the evidence base for those offerings. Very few units told of full proactive care pathways for people with autism. This is major failing towards people with autism.

A further concern is the small but noteworthy minority of units unable to offer any autism-specific adaptations. This further establishes that people with autism remain vulnerable to the vagaries of local commissioning. Given the above situation it is not surprising that people with autism are more likely to be delayed in discharge and more prone to segregation. This is a vicious circle as it further perpetuates institutionalisation and increases community breakdown. It is imperative that issues such as delayed discharge and segregation be seen in continuum with the unit type, staff skill set, and assessments and processes in place for supporting in-patients with autism. This survey highlights multiple issues on clinical, training, policy and research matters.

### Implications for the patient

Our co-author, E.S., representing a national charity, who helped design the study, shares his perspective in response to the results of this study:

‘Autistic people have the right to mental health care that meets their needs. These findings illustrate what many in the autism community suspected: that in-patient services lack clear guidance on how to best support autistic people in their care. To enable that guidance to be developed, the Government and NHS needs to direct resources towards closing fundamental gaps in the evidence base. These findings highlight just how little clarity there is on the effectiveness and safety of different approaches to providing in-patient care for autistic people. The initial insights from this study – on differences in environmental adaptations, staffing and intervention models – provide possible starting points for further exploration. Reliably testing which of those interventions and adaptations are effective, and under what circumstance, would help the NHS take a solid step towards developing evidenced clinical pathways from admission through to discharge.’

### Implications for clinical practice

There is an urgent need to establish and incorporate an evidence-based clinical pathway from admission to discharge for all in-patients with autism across all psychiatric settings. The pathway needs to include all essential elements from a biopsychosocial perspective to support the assessment and treatment of the emotional and behavioural needs of people with autism. Suitable workable and valid clinical outcome measures can help compare and improve clinical delivery.

### Implications for training

Focus has to be on ensuring the care of people with autism is led by a well-trained and informed staff team irrespective of their individual clinical discipline. Every unit open to admitting people with autism needs to meet high levels of formal training standards on autism care. A minimum standard training framework co-produced with patients would be an important step forward. Using experts-by-experience in the training of staff would be novel, empathetic and deliver better outcomes.^17^ It is expected that there will be basic autism training and more skilled professional autism competencies (see https://www.hee.nhs.uk/our-work/learning-disability/oliver-mcgowan-mandatory-training-learning-disability-autism). Recognition of a suitable blend of basic autism awareness training and a more developed competency based training for those who work closely with autistic peopleshould be suitably implemented.

### Implications for policy

The levels of ambiguity, heterogeneity and different skill sets of staff found in different types of units identified in our study is very concerning. In addition, the significant gap in support for people with autism between intellectual disability specialised units versus general units needs addressing through suitable policy measures. The improvements outlined for clinical practice and training need to be encapsulated into suitable policy initiatives. There needs to be an open dialogue on how to ensure proactive commissioning to facilitate seamless in-patient assessment and treatment to prevent distress and trauma when admissions are needed. It is also important to explore how support for people with autism from healthcare, social care and the voluntary sector in the community can be enhanced to minimise admissions and facilitate early discharge. Joint commissioning that focuses on the timely and individualised support of people with autism may be a way of achieving this.

### Implications for research

Lack of research means there is an absence of the high-quality evidence that is required in order to gain a greater understanding of the best treatment approaches and a full understanding of the experiences of people with autism in in-patient settings. A larger national study proactively looking to capture clinical outcomes and the patient experience could lead to improved understanding and insight into current issues and concerns.

## Data Availability

The data that support the findings of this study are available from the corresponding author upon reasonable request.

## Supplementary material

For supplementary material accompanying this paper visit http://dx.doi.org/10.1192/bjo.2021.58. click here to view supplementary material

## References

[1] Tromans S, Chester V, Kiani R, Alexander R, Brugha T. The prevalence of autism spectrum disorders in adult psychiatric inpatients: a systematic review. Clin Pract Epidemiol Ment Heal 2018; 14: 177–87. 10.2174/1745017901814010177 PMC6118035 30197663

[2] Mandell DS, Lawer LJ, Branch K, Brodkin ES, Healey K, Witalec R, Prevalence and correlates of autism in a state psychiatric hospital. Autism 2011; 16: 557–67. 21846667 10.1177/1362361311412058

[3] National Autistic Society (NAS). Beyond Transforming Care, What Needs To Change? NAS, 2018 (https://www.basw.co.uk/system/files/resources/Beyond%20Transforming%20Care%20report-final-version.pdf).

[4] Emerson E, Baines S. The Estimated Prevalence of Autism among Adults with Learning Disabilities in England. The Learning Disabilities Observatory, 2010 (http://www.wenurses.eu/MyNurChat/archive/LDdownloads/vid_8731_IHAL2010-05Autism.pdf).

[5] Association of Directors of Adult Social Services (ADASS); Local Government Association (LGA); NHS England. Supporting People with a Learning Disability and/or Autism who Display Behaviour that Challenges, Including those with a Mental Health Condition. ADASS, LGA, NHS England, 2015. (https://www.england.nhs.uk/wp-content/uploads/2015/10/service-model-291015.pdf).

[6] Lai MC, Cassee C, Besney R, Bonato S, Hull L, Mandy W, Prevance of co-occurring mental health diagnosis in the autism population: a systematic review and meta-analysis. Lancet Psychiatry 2019; 6: 819–29. 31447415 10.1016/S2215-0366(19)30289-5

[7] Royal College of Psychiatrists. The Psychiatric Management of Autism in Adults - College Report CR22*8*. Royal College of Psychiatrists, 2020 (https://www.rcpsych.ac.uk/docs/default-source/improving-care/better-mh-policy/college-reports/college-report-cr228.pdf?sfvrsn=c64e10e3_2).

[8] Cassidy S, Bradley P, Robinson J, Allison C, McHugh M, Baron-Cohen S. Suicidal ideation and suicide plans or attempts in adults with Asperger's syndrome attending a specialist diagnostic clinic: a clinical cohort study. Lancet Psychiatry 2014; 1: 142–7. 26360578 10.1016/S2215-0366(14)70248-2

[9] UK Government. *The Autism Act*. UK Government, 2009. UK Government, 2009 ( http://www.legislation.gov.uk/ukpga/2009/15/contents).

[10] Department of Health and Social Care. Statutory Guidance for Local Authorities and NHS Organisations to Support Implementation of the Adult Autism Strategy. UK Government, 2015 (https://assets.publishing.service.gov.uk/government/uploads/system/uploads/attachment_data/file/422338/autism-guidance.pdf).

[11] Maloret P, Scott T. Don't ask me what's the matter, ask me what matters: acute mental health facility experiences of people living with autism spectrum conditions. J Psychiatr Ment Health Nurs 2018; 25: 49–59. 29078024 10.1111/jpm.12438

[12] Department of Health. Mental Health Act 1983: Code of Practice. UK Government, 2015 (https://assets.publishing.service.gov.uk/government/uploads/system/uploads/attachment_data/file/435512/MHA_Code_of_Practice.PDF).

[13] McGill P, Murphy G, Kelly-Pike A. Frequency of use and characteristics of people with intellectual disabilities subject to physical interventions. J Appl Res Intellect Disabil 2009; 22: 152–8.

[14] David M, Hannah M. Examining the experiences and quality of life of patients with an autism spectrum disorder detained in high secure psychiatric care. Adv Autism 2017; 3: 3–14.

[15] Murphy D, Bush EL, Puzzo I. Incompatibilities and seclusions among individuals with an autism spectrum disorder detained in high secure psychiatric care. J Intell Disabil Offending Behav 2017; 8: 188–200.

[16] Sourander A, Ellilä H, Välimäki M, Piha J. Use of holding, restraints, seclusion and time-out in child and adolescent psychiatric in-patient treatment. Eur Child Adolesc Psychiatry 2002; 11: 162–7. 12444425 10.1007/s00787-002-0274-2

[17] Alexander RT, Langdon PE, O'Hara J, Howell A, Lane T, Tharian R, Psychiatry and neurodevelopmental disorders: experts by experience, clinical care and research. Br J Psychiatry 2021; 218(1): 1–3. 33541475 10.1192/bjp.2020.237

