# Supplementary material for: Current practice and adaptations being made for people with autism admitted to in-patient psychiatric services across the UK
Source: BJPsych Open. 2021 May 14;7(3):e102. doi: 10.1192/bjo.2021.58 (PMC8161595; doi:10.1192/bjo.2021.58)
Supplement: Supplementary file 1 [file bjosup.zip › supplementary information 1.pdf]

Inpatient care for autistic people with mental health problems- how are we doing?

Aim of this short survey (takes only about 5 minutes) is to understand current provision in this area and to identify any structural and process adaptations that are being made, with a view to sharing best practice locally and nationally.

THANK YOU FOR YOUR TIME.

basic information

The following questions are going to ask you for some basic demographic information. The information will not compromise your anonymity

1. 2. What is the nature of the inpatient unit you support? Please tick all suitable.

Check all that apply.

- [ ] Adult Learning Disability (LD) Unit
- [ ] Forensic Unit
- [ ] Forensic LD unit
- [ ] General Psychiatric Unit (accepting autistic people)
- [ ] General psychiatric intensive care unit (accepting autistic people)
- [ ] Medium/high secure LD unit

Other: [ ] ______________________________

2. 3. Where is the inpatient unit based? Please put first part of the postcode to identify any duplicate entries on the same inpatient unit. This will be made anonymous in the analysis.

____

3. What is the funding stream of your inpatient setting

Mark only one oval.

- NHS
- Private
- Mixed

4. What is the inpatient assessment and treatment provision generally for people with LD and acute mental health problems within your organisation? (tick all relevant)

Check all that apply.

- [ ] Have a local specialist assessment and treatment unit for people with LD
- [ ] Have access to assessment and treatment beds within general adult mental health inpatient units
- [ ] Block commissioning of private sector beds for assessment and treatment of people with LD
- [ ] Spot purchase of assessment and treatment beds as required for people with LD

Other: [ ] ____________________

5. Approximate percentage of autistic people compared to the total inpatient numbers who have utilized the unit over the last year

Mark only one oval.

- <10%
- 10-25%
- 25 -50%
- greater than 50%
- not sure

Section 2 Adaptations for Autism Spectrum Disorder (ASD)

This sections looks at specific issues of ASD in your inpatient population

6. Does your staff group supporting the inpatient unit have any ASD specific expertise – tick as many as appropriate

Check all that apply.

- [ ] Psychiatry (formal training such as DISCO or ADOS or other similar approaches)
- [ ] SALT with specialist skills in assessment of communication needs in autistic people
- [ ] OT expertise in sensory assessments and designing therapeutic activities for autistic people
- [ ] Nursing team –specialist autism training; specialist autism-related needs assessments
- [ ] Psychologist with ASD expertise

Other: [ ] ______________________

7. Are discharge delays less, about the same, or more in autistic people compared to other patient groups in the unit

Mark only one oval.

- ( ) Less likely to have discharge delays than other groups
- ( ) about the same
- ( ) more likely to have discharge delays than other groups
- ( ) cannot quantify

8. On admission, do you construct care plans specifically designed to support Autistic people?

____

9. Before or after admitting a person with known or suspected ASD, do you undertake any specific assessments or formally consider any adaptations such as - (tick all relevant)

Check all that apply.

- [ ] Sensory assessments
- [ ] Likes / dislikes
- [ ] Coping strategies including any tools in place for supporting the individual
- [ ] Communication assessments
- [ ] None of the above

Other: [ ] ______________________

10. Does your unit have any specific environmental or sensory adaptations for autistic people? (tick as many appropriate)

Check all that apply.

- [ ] Open access low stimulus areas
- [ ] On request low stimulus areas
- [ ] Scheduled access low stimulus areas
- [ ] Lighting adaptations
- [ ] The ability to adapt meal plans to sensory requirements
- [ ] Noise adaptations (e.g. silent alarms, sound proofing)
- [ ] None of the above

Other: [ ] ______________________

11. In the past year, have you had any inpatients with an ASD (suspected/diagnosed) in long term segregation?

Mark only one oval.

- ( ) Yes
- ( ) No
- ( ) Not applicable

12. In your experience how likely are autistic people to be secluded than the other inpatients?

Mark only one oval.

- less likely
- same
- more likely
- significantly more likely
- Not sure

13. Does your unit have any specific communication tools designed to support autistic people?

Check all that apply.

- [ ] Visual signage / orientation tools
- [ ] Visual timetables
- [ ] Visual help / cue cards
- [ ] Access to social stories, when required
- [ ] None of the above

Other: [ ] ______________________

14. Do you have any other tools or strategies designed for specifically to support autistic people? Please give details.

15. Do you have a specific protocol to admit, identify, diagnose, support, manage and discharge an autistic person in your unit?

Mark only one oval.

- ( ) Yes
- ( ) No
- ( ) Don't know

16. Any other comments relevant to this survey?

____

____

____

____

____

Thank you very much for your time!

This content is neither created nor endorsed by Google.

Google Forms
